# Oral acute graft‐versus‐host disease

**DOI:** 10.1002/jha2.1033

**Published:** 2024-10-20

**Authors:** Farzad Teymouri, Gale Sebastian, Hakan Gem, Samuel S. Minot, Armin Rashidi

**Affiliations:** ^1^ Department of Internal Medicine Eisenhower Health Rancho Mirage California USA; ^2^ Department of Oral Medicine University of Washington and Fred Hutchinson Cancer Center Seattle Washington USA; ^3^ Data Core, Shared Resources Fred Hutchinson Cancer Center Seattle Washington USA; ^4^ Clinical Research Division Fred Hutchinson Cancer Center Seattle Washington USA; ^5^ Division of Medical Oncology, Department of Medicine University of Washington Seattle Washington USA

**Keywords:** GVHD, microbiota, oral, sequencing

## Abstract

Oral acute graft‐versus‐host disease (aGVHD) is rare and with no diagnostic criteria. We report a case of oral aGVHD with three clinical phases. A self‐limited prodrome of largely subjective oral symptoms was followed by concurrent oral and upper gastrointestinal aGVHD. Six months after transplantation, the patient was diagnosed with severe oral and upper gastrointestinal chronic GVHD. We compared the salivary microbiota of our patient at the time of diagnosis of aGVHD with 50 contemporaneous transplant recipients and found no evidence for oral microbiota involvement in pathogenesis. This in‐depth N‐of‐1 analysis reveals novel aspects of oral aGVHD pathogenesis.

1

Graft‐versus‐host disease (GVHD)—the clinical phenotype of an alloimmune attack from the graft against host epithelial tissues—is a challenging complication of allogeneic hematopoietic cell transplantation (alloHCT). Despite prophylaxis, GVHD develops in nearly half of the alloHCT recipients and is a leading cause of morbidity and mortality after transplant. GVHD is classified as acute or chronic based on clinical manifestations and time of onset [1]. Acute GVHD (aGVHD) is further divided into classic (onset within 100 days after HCT) and late‐onset (onset between days +100 and +180). The three main organs involved by aGVHD are the skin, liver, and gastrointestinal tract. Other organs such as kidneys, lungs, eyes, and the central nervous system are only rarely involved. In contrast, chronic GVHD (cGVHD) can affect virtually any organ. While oral cGVHD is common and has established diagnostic criteria [2], the mouth is not a classical target organ in aGVHD. Nevertheless, most transplant physicians have encountered a few highly unusual cases of oral GVHD that do not follow the typical clinical patterns of oral cGVHD and despite having some degree of similarity to oral cGVHD, seem to represent a separate entity. We are aware of only one published series of oral aGVHD [3]. Due to the rarity of oral aGVHD, its pathogenesis and optimal treatment are unknown. Here, we report the unique clinical course of a patient with oral aGVHD. In addition, as our recent analyses suggested a role for the oral microbiota in oral cGVHD pathogenesis [4, 5], we hypothesized that oral dysbiosis may also mediate the pathogenesis of oral aGVHD. We tested this hypothesis by comparing the oral microbiota of our patients to 50 contemporaneous alloHCT recipients.

A 41‐year‐old Ethiopian man with a past medical history of pulmonary tuberculosis was referred for alloHCT for blastic plasmacytoid dendritic cell neoplasm in complete remission after receiving the HyperCVAD regimen (mods 1A through 2A). The skin, lymph nodes, bone marrow, and blood were originally involved with the disease. Standard pretransplant work up was unremarkable. Baseline oral examination, performed by an oral medicine specialist, showed widespread heavy plaque and calculus accumulation in the setting of chronic generalized periodontal disease and severe localized periodontal bone loss in one tooth. Full mouth scaling and root planning, topical minocycline, and a tooth extraction were performed prior to conditioning. Transplant conditioning included cyclophosphamide and total body irradiation (12 Gy). Peripheral blood hematopoietic cells from a human leukocyte antigen‐matched sibling were used for transplantation. GVHD prophylaxis included tacrolimus and methotrexate. Levofloxacin during the period of severe neutropenia, trimethoprim‐sulfamethoxazole, and valacyclovir were used for infectious prophylaxis. The patient developed grade 3 conditioning‐related oral mucositis, requiring intravenous narcotics for pain management and total parenteral nutrition for nutritional support for a week. His only infectious complication in the early post‐transplant period was a urinary tract infection at day +30, treated with levofloxacin for 1 week.

Twenty‐eight days after alloHCT, the patient experienced a gradual recurrence of anorexia, nausea, and early satiety that he had experienced earlier after transplantation but had almost resolved. A clinical diagnosis of possible upper gastrointestinal aGVHD was made, but due to the mild nature of symptoms, the patient opted against immunosuppressive treatment and was managed with supportive care. These symptoms continued for about 3 weeks before spontaneous improvement to baseline levels. Approximately 35 days after alloHCT, and 3 weeks after complete resolution of conditioning‐related mucositis and while upper gastrointestinal symptoms were at peak, the patient developed acute‐onset burning oral pain (buccal mucosa, tongue, and throat) and a subjective feeling of tongue swelling over a few days; the pain interfered with oral intake. Oral examination, partly compromised by limited mouth opening due to pain, revealed cracked lips, buccal mucosa tenderness, a white exudate on the tongue and palate, and patchy redness at the oral mucosa. These symptoms improved over the following week with no intervention. Oral examination at approximately 50 days after alloHCT showed erythema (E1), hyperkeratosis, and fine lichenoid changes (L2) involving the buccal mucosa, soft palate, tongue, and gingiva (Figure 1A–C). Infection was ruled out by a lesional swab. A diagnosis of oral aGVHD was established. Treatment with topical dexamethasone resulted in resolution of symptoms. At approximately 6 months after transplant, the patient experienced a recurrence of oral symptoms, including cracked lips, pain/burning sensation, limited oral intake, dry mouth, and limitation in mouth opening. Examination revealed moderate to severe erythema (E2), pseudomembranous ulcerations (U3) at the buccal mucosa, and thick lichenoid striations (L3) on bilateral buccal mucosa, all surfaces of the tongue, hard and soft palate, gingiva, upper and lower labial mucosa, and mucoceles on the soft palate (Figure 1D–F). With a diagnosis of oral cGVHD, topical dexamethasone was reinitiated. An esophagogastroduodenoscopy (Figure 1G–I) and biopsy performed for work up of new dysphagia showed findings consistent with GVHD of the esophagus (necrotizing and severe), stomach (moderate and with glandular destruction), and duodenum (mild and with rare apoptotic cells), with no evidence of infection. A diagnosis of upper gastrointestinal cGVHD was established.

**FIGURE 1:**
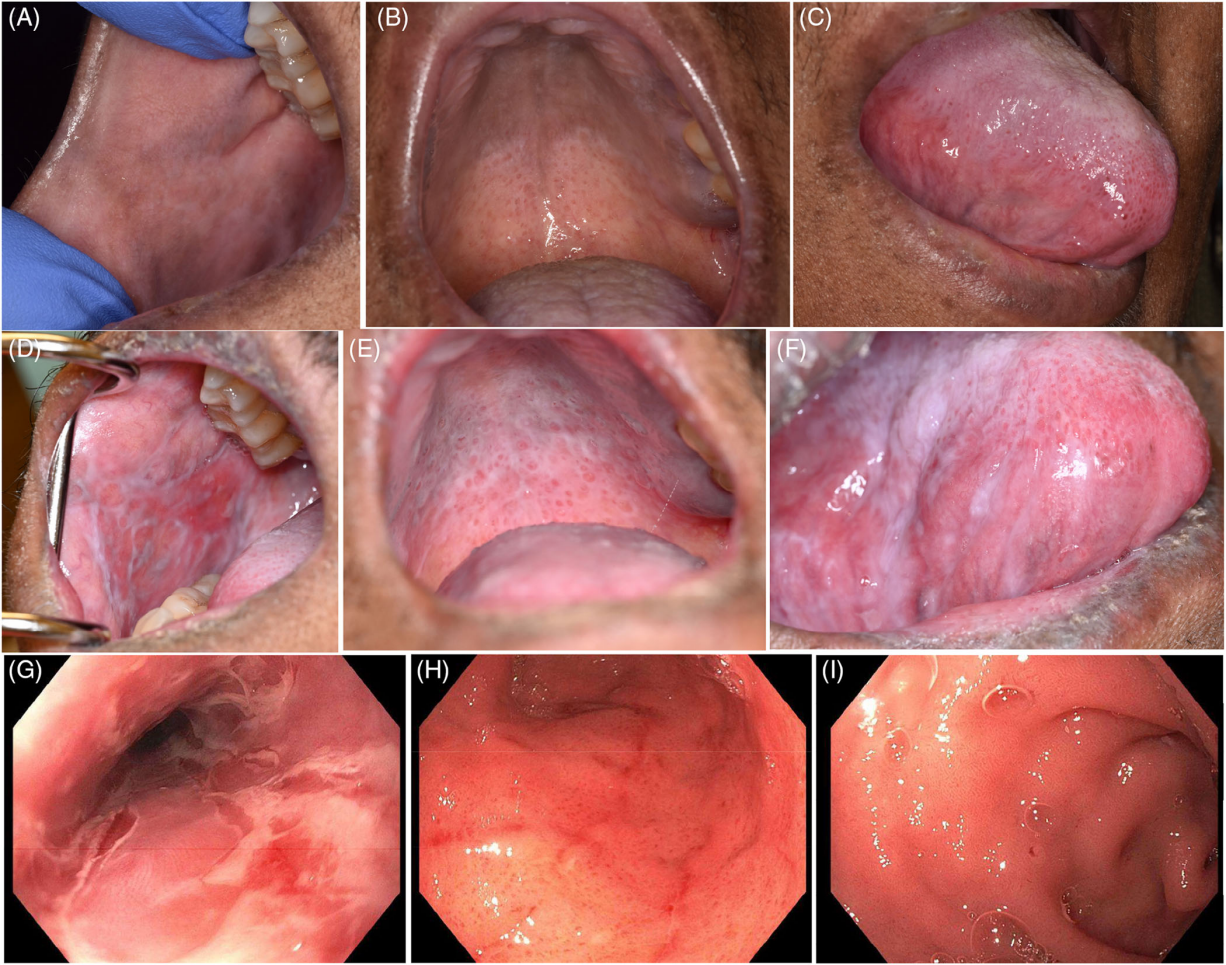
Findings on physical examination. Panels (A–C) show findings on oral examination at the time of oral acute graft‐versus‐host disease (GVHD) diagnosis. Panels (D–F) show findings on oral examination at the time of oral chronic GVHD diagnosis. Panels (G–I) show findings on esophagogastroduodenoscopy at the time of upper gastrointestinal chronic GVHD diagnosis. (A) Lichenoid striations involving ∼50% of the surface area of the buccal mucosa. (B) Lichenoid striations in a reticular pattern on the soft palate. (C) Lichenoid striations involving the lateral surface of the tongue. (D) Thick lichenoid striations involving almost 80% of the surface of the buccal mucosa, with moderate erythema, atrophy, and pseudomembranous ulcerations. (E) Thick lichenoid striations involving nearly the entire hard and soft palate, with moderate erythema and scattered mucoceles. (F) Thick lichenoid striations involving the lateral surface of the tongue, with mild to moderate erythema. (G) Esophagitis with necrosis. (H) Gastritis. (I) Duodenitis.

A salivary sample was obtained at the time of oral aGVHD diagnosis as part of the Fred Hutchinson Cancer Center (FHCC)’s IRB‐approved biorepository protocol focused on the microbiota in patients with cancer. The patient provided written informed consent. As the comparator group, we included 87 salivary samples collected from 50 adult alloHCT recipients as part of another FHCC IRB‐approved noninterventional study focused on the oral microbiota in alloHCT recipients. All patients provided written informed consent. Three criteria were used to select the samples for comparison: (*i*) time of collection relative to alloHCT within a ±28‐day window of the collection timepoint of our index case, (*ii*) not more than two samples from the same patient, and (*iii*) samples from the same patient at least 28 days apart. Methods for sequencing and bioinformatic analysis are detailed in the Supporting Information.

Patient characteristics are summarized in Table S1 and peritransplant antibiotic exposures are shown in Figure 2A. Microbiota diversity of the index sample was almost equal to the median diversity of the other samples (Figure 2B). In principal coordinates analysis, the index sample was well within the mix of all other samples, indicating no distinct microbiota signature (Figure 2C) and consistent with a visual examination of taxonomic distributions (Figure 2D). The Aitchison distance between the index case and other samples was similar to all pairwise distances between the other samples (Wilcoxon's *p* = 0.24), indicating no overall compositional differences between the index case and other samples. These findings indicated no evidence for a microbiome‐mediated process.

**FIGURE 2:**
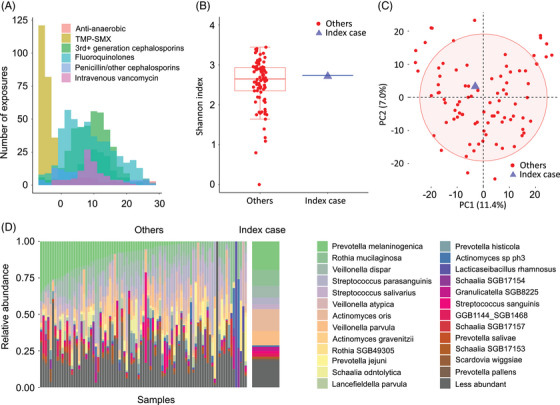
Salivary microbiota signature of oral acute graft‐versus‐host disease. (A) Antibacterial antibiotic exposures between days −7 and +28. The six most commonly used antibiotics are shown. TMP‐SMX, trimethoprim‐sulfamethoxazole. (B) Alpha diversity as measured by the Shannon's index. The box shows the median (horizontal middle line) and interquartile range. Whisker lines indicate nonoutlier maximum and minimum values. A small jitter is included for better visualization. (C) Beta diversity and ordination visualized by principal coordinate analysis. Aitchison distance (using centered log‐ratio species abundances) was used to quantify the overall compositional difference between samples. The first two principal coordinates (PC1 and PC2) are shown. Numbers in parentheses indicate percent variation explained by the corresponding axis. The closer the two samples, the more similar their microbiome compositions. An 80% ellipse is shown. (D) Relative abundances of the 25 most abundant species among all samples.

The two most common noninfectious oral complications of alloHCT are: (*i*) oral mucositis due to the conditioning regimen and (*ii*) oral cGVHD. Oral mucositis usually occurs 7–10 days after the conditioning regimen and presents as painful, ulcerative, erythematous lesions in the oral mucosa. Oral cGVHD is a late complication, with a median onset of 7 months after alloHCT and presenting with dry mouth and mucosal changes including erythema, lichenoid changes, and ulcers [6]. In contrast to oral mucositis and oral cGVHD, oral aGVHD is rare. The largest published report is a retrospective study of 2578 alloHCT recipients, 21 (0.8%) of whom developed oral aGVHD [3] at a median of 35 days after HCT. The mouth was the only involved organ in five of these patients. The most common oral findings were erythema, edema, pseudomembranous ulcerations, and mucosal atrophy. The sites most frequently involved were the buccal mucosa (90%), tongue (86%), labial mucosa (76%), and palatal mucosa (71%). Lichenoid changes are less prominent in oral aGVHD than oral cGVHD [3, 7].

As no specific symptom or sign distinguishes oral aGVHD from cGVHD, early onset and the concurrent involvement of other organs by aGVHD are often used to diagnose oral aGVHD. Complete resolution of mucositis before the onset of symptoms in our patient eliminated conditioning‐related toxicity as the diagnosis, while the onset of symptoms within 2 months of alloHCT in parallel with upper gastrointestinal aGVHD favored a diagnosis of oral aGVHD. After an initial response to topical steroids, our patient presented with symptoms and signs (including lichenoid changes) that were much more prominent than the original presentation, leading to a diagnosis of oral cGVHD. Although aGVHD seems to increase the risk of subsequent cGVHD in general [8, 9], whether the same relationship exists for oral GVHD is unknown.

The diagnostic criteria for oral cGVHD capture a great majority of patients with oral GVHD. However, distinguishing between oral cGVHD and the rare oral aGVHD is not an intended goal for these criteria. Therefore, careful attention to the detailed clinical history and evolution of signs/symptoms over time is key to the correct diagnosis of oral aGVHD. Finding one or more features of oral cGVHD in a case that would otherwise not fit the expected presentation or behavior of oral cGVHD should not lead to the diagnosis of oral cGVHD. The mild lichenoid changes found on physical examination in our patient well illustrate this scenario. A three‐stage clinical course was apparent in our patient: (i) a self‐limited prodrome of largely subjective oral symptoms which likely triggered downstream events, (ii) steroid‐responsive oral aGVHD, and (iii) severe oral and upper gastrointestinal cGVHD. Stages (i) and (ii) occurred while upper gastrointestinal aGVHD was present. Although time of onset per se does not distinguish between aGVHD and cGVHD, concurrent presence of other organ aGVHD and near absence of cGVHD findings in stages (i) and (ii) confirmed the diagnosis of oral aGVHD.

The pathogenesis of oral aGVHD is elusive and its rarity makes research on this topic challenging. The extent to which the mechanisms of acute and chronic oral GVHD overlap is also unclear, but the similarity of signs and symptoms makes some degree of overlap likely. Our patient's prompt response to topical steroids indicates an inflammatory component. A large repository of oral samples in alloHCT patients allowed us to examine whether the oral microbiota in our patient had unusual features, thereby suggesting a microbiota‐mediated process. Shotgun metagenomic sequencing argued against microbiota involvement. Histopathological examination, proteomics, and metabolomics in future cases may help reveal the pathogenetic mechanisms of oral aGVHD.

## AUTHOR CONTRIBUTIONS

Armin Rashidi and Hakan Gem designed the research study. All authors performed the research. Armin Rashidi analyzed the data. Armin Rashidi and Farzad Teymouri wrote the paper.

## CONFLICT OF INTEREST STATEMENT

Armin Rashidi has received consulting fees from Seres Therapeutics and serves as a paid member of an Emmes Data and Safety Monitoring Board, both outside of the scope of the present study.

## ETHICS STATEMENT

The authors have confirmed ethical approval statement is not needed for this submission.

## PATIENT CONSENT STATEMENT

The authors have confirmed patient consent statement is not needed for this submission.

## CLINICAL TRIAL REGISTRATION

The authors have confirmed clinical trial registration is not needed for this submission.

## Supporting information



Supporting Information

Supporting Information

## Data Availability

Taxonomic tables related to data reported in this paper are available from the corresponding author upon email request.
