# Supplementary material for: Oral acute graft‐versus‐host disease
Source: EJHaem. 2024 Oct 20;5(6):1290–4. doi: 10.1002/jha2.1033 (PMC11647690; doi:10.1002/jha2.1033)
Supplement: Supplementary file 1 — Supporting Information [file JHA2-5-1290-s002.docx]

**Supplementary Methods**

DNA was extracted using the ZymoBIOMICS®-96 MagBead DNA Kit (Zymo Research, Irvine, CA). Sequencing libraries were prepared using the Illumina® DNA Library Preparation Kit (Illumina, San Diego, CA) following the manufacturer’s protocol and with unique dual-index 10 bp barcodes with Nextera® adapters. All libraries were pooled in equal abundance and the final pool was quantified using qPCR and TapeStation® (Agilent Technologies, Santa Clara, CA). The final library was sequenced on an Illumina NovaSeq 6000 using a S2-300 flow cell and a PE150 configuration. The target sequencing depth was 20M read pairs per sample. Raw paired-end metagenomic sequence reads were quality-processed using the integrated pipeline provided in KneadData v.0.12.0. This sequence-level procedure included two main steps: (*i*) removal of reads mapped to the human reference genome GRCh37 (hg19) using Bowtie2 v.2.4.5 [(Langmead and Salzberg 2012)](https://paperpile.com/c/xQp6pc/QkNOC) and (*ii*) removal of adapter sequences and low-quality reads using Trimmomatic v.0.39 [(Bolger, Lohse, and Usadel 2014)](https://paperpile.com/c/xQp6pc/eC7TA) with default settings. Output files consisting of surviving paired and orphan reads were concatenated and used as input to MetaPhlAn4 [(Blanco-Míguez et al. 2023)](https://paperpile.com/c/xQp6pc/yN3mH). MetaPhlAn4 with default parameters was used for species-level taxonomic assignment. MetaPhlAn4 uses a set of species-level genome bins (SGBs) [(Pasolli et al. 2019)](https://paperpile.com/c/xQp6pc/wvtNm) as primary taxonomic units and accurately profiles their presence and abundance in metagenomes. The latest version (version 4) of MetaPhlAn uses a database containing ~5.1 million unique clade-specific marker genes for 21,978 existing SGBs and 4,992 yet-to-be-characterized SGBs (defined solely based on metagenome-assembled genomes). Within-sample diversity (i.e. alpha diversity) was quantified by Shannon index [(Shannon and Weaver 1949)](https://paperpile.com/c/xQp6pc/3gsid). Between-sample diversity (i.e. beta diversity) was quantified by Aitchison distance [(Aitchison et al. 2000)](https://paperpile.com/c/xQp6pc/7o6xs) and visualized by principal coordinate analysis.

**References**

[Aitchison, J., C. Barceló-Vidal, J. A. Martín-Fernández, and V. Pawlowsky-Glahn. 2000. “Logratio Analysis and Compositional Distance.” *Mathematical Geology* 32 (3): 271–75.](http://paperpile.com/b/xQp6pc/7o6xs)

[Blanco-Míguez, Aitor, Francesco Beghini, Fabio Cumbo, Lauren J. McIver, Kelsey N. Thompson, Moreno Zolfo, Paolo Manghi, et al. 2023. “Extending and Improving Metagenomic Taxonomic Profiling with Uncharacterized Species Using MetaPhlAn 4.” *Nature Biotechnology* 41 (11): 1633–44.](http://paperpile.com/b/xQp6pc/yN3mH)

[Bolger, Anthony M., Marc Lohse, and Bjoern Usadel. 2014. “Trimmomatic: A Flexible Trimmer for Illumina Sequence Data.” *Bioinformatics*  30 (15): 2114–20.](http://paperpile.com/b/xQp6pc/eC7TA)

[Langmead, Ben, and Steven L. Salzberg. 2012. “Fast Gapped-Read Alignment with Bowtie 2.” *Nature Methods* 9 (4): 357–59.](http://paperpile.com/b/xQp6pc/QkNOC)

[Pasolli, Edoardo, Francesco Asnicar, Serena Manara, Moreno Zolfo, Nicolai Karcher, Federica Armanini, Francesco Beghini, et al. 2019. “Extensive Unexplored Human Microbiome Diversity Revealed by Over 150,000 Genomes from Metagenomes Spanning Age, Geography, and Lifestyle.” *Cell* 176 (3): 649–62.e20.](http://paperpile.com/b/xQp6pc/wvtNm)

[Shannon, Claude Elwood, and Warren Weaver. 1949. “The Mathematical Theory of Communication. Univ.” *Illinois Press, Urbana, I* 11:117.](http://paperpile.com/b/xQp6pc/3gsid)
