# Supplementary material for: Oral acute graft‐versus‐host disease
Source: EJHaem. 2024 Oct 20;5(6):1290–4. doi: 10.1002/jha2.1033 (PMC11647690; doi:10.1002/jha2.1033)
Supplement: Supplementary file 2 — Supporting Information [file JHA2-5-1290-s001.docx]

**Supplementary Table S1:** Patient characteristics

| Total, N | 50 |
| --- | --- |
| Sex  Male  Female | 25  25 |
| Age at transplantation, yrs | 43 (20-60) |
| Underlying disease  Acute leukemia  MDS/MPN  Non-Hodgkin lymphoma | 39  7  4 |
| Conditioning regimen  Myeloablative  High-dose TBI-based  Bu/Cy  Flu/Bu4  Flu/Cy/Thiotepa/TBI (4 Gy)  Reduced intensity  Flu/Mel/TBI (2-4 Gy)  Flu/Treosulfan/TBI (2 Gy) | 20  11  11^1^  4  2  2 |
| Donor type  HLA-matched sibling  HLA-matched unrelated  HLA-haploidentical  Cord blood  HLA-mismatched unrelated (1 antigen) | 12  26  4  7  1 |
| Graft source  Peripheral blood  Cord blood | 43  7 |
| GVHD prophylaxis  Tacrolimus/Methotrexate  PTCy-based  Cyclosporine/MMF | 31  12  7 |

^1^Four patients received additional TBI (4 Gy)

Bu: busulfan; Cy: cyclophosphamide; Flu: fludarabine; GVHD: graft-versus-host prophylaxis; HLA: human leukocyte antigen; MDS: myelodysplastic syndromes; MMF: mycophenolate mofetil; MPN: myeloproliferative neoplasms; PTCy: post-transplantation cyclophosphamide; TBI: total body irradiation
